# Changes in Variable Number of Tandem Repeats in ‘*Candidatus* Liberibacter asiaticus’ through Insect Transmission

**DOI:** 10.1371/journal.pone.0138699

**Published:** 2015-09-24

**Authors:** Hiroshi Katoh, Hiromitsu Inoue, Toru Iwanami

**Affiliations:** 1 NARO Institute of Fruit Tree Science, Fujimoto 2-1, Tsukuba, Ibaraki 305-8605, Japan; 2 Kuchinotsu Citrus Research Station, NARO Institute of Fruit Tree Science, Minami-shimabara, Nagasaki 859–2501, Japan; USDA/ARS, UNITED STATES

## Abstract

Citrus greening (huanglongbing) is the most destructive citrus disease worldwide. The disease is associated with three species of ‘*Candidatus* Liberibacter’ among which ‘*Ca*. Liberibacter asiaticus’ has the widest distribution. ‘*Ca*. L. asiaticus’ is commonly transmitted by a phloem-feeding insect vector, the Asian citrus psyllid *Diaphorina citri*. A previous study showed that isolates of ‘*Ca*. L. asiaticus’ were clearly differentiated by variable number of tandem repeat (VNTR) profiles at four loci in the genome. In this study, the VNTR analysis was further validated by assessing the stability of these repeats after multiplication of the pathogen upon host-to-host transmission using a ‘*Ca*. L. asiaticus’ strain from Japan. The results showed that some tandem repeats showed detectable changes after insect transmission. To our knowledge, this is the first report to demonstrate that the repeat numbers VNTR 002 and 077 of ‘*Ca*. L. asiaticus’ change through psyllid transmission. VNTRs in the recipient plant were apparently unrelated to the growing phase of the vector. In contrast, changes in the number of tandem repeats increased with longer acquisition and inoculation access periods, whereas changes were not observed through psyllid transmission after relatively short acquisition and inoculation access periods, up to 20 and 19 days, respectively.

## Introduction

Citrus greening (huanglongbing) is a devastating citrus disease globally. The disease was first reported in China in the early 20^th^ century, and it was known as yellow shoot disease in this region [1]. By the 1920s, diseases similar to yellow shoot disease were recorded in Taiwan (likubin or drooping disease) [2] and India (citrus dieback) [3]. In the 1940s, the disease was initially recorded in Indonesia and was described as vein phloem degeneration [4]. Since then, citrus greening has been reported in Japan and South Africa [5, 6]. In the western hemisphere, citrus greening was first reported in Sao Paulo, Brazil [7], and then in Florida, USA [8]. After the initial findings, the disease has apparently spread all over the Caribbean, North and Central American countries in a relatively short period [9, 10, 11, 12, 13, 14, 15].

The causal agents are phloem-limited gram-negative bacteria. Thus far, three species of this organism have been identified; ‘*Ca*. Liberibacter africanus’ is mainly found in Africa, ‘*Ca*. Liberibacter americanus’ is found in Brazil [16], and ‘*Ca*. Liberibacter asiaticus’ is widely distributed in Asian countries as well as in the Americas. The pathogens are transmitted by the African citrus psyllid, *Trioza erytreae* (Del Guercio), in Africa [17] and the Asian citrus psyllid, *Diaphorina citri* Kuwayama, in Asia and the Americas [18]. The disease also spreads with contaminated plant material used for the propagation of nursery plants.

Methods for distinguishing bacterial isolates are important for epidemiological analyses to elucidate the genetic structure of microbial populations. Variable number of tandem repeat (VNTR) markers, also known as microsatellites or simple sequence repeats, are tandem repetitive DNA sequences with repeated motifs of 2–6 bp or more [19]. VNTRs in bacterial DNA have been used as markers for differentiating and subtyping strains of several bacterial species, including ‘*Ca*. L. asiaticus’ [20, 21, 22]. In a previous study, Katoh et al. [21] selected four highly polymorphic VNTR loci and these VNTR markers were used to estimate the genetic diversity and population structures of ‘*Ca*. L. asiaticus’ in Japan, Taiwan, India, Indonesia, Timor-Leste, Papua New Guinea, and Florida [21, 22]. Using these VNTR markers at four loci, Matos et al. [23] also performed an extended analysis of ‘*Ca*. L. asiaticus’ populations in several countries, including the Caribbean, Central America regions, and Mexico.

Matos et al. [23] also examined the stability of VNTRs in the four loci of the ‘*Ca*. L. asiaticus’ genome after transmission of the pathogen in citrus and psyllid hosts. It was shown that the profiles do not change upon passage of the pathogen in citrus and psyllid hosts as well as after it has remained within a host for over five years. However, the number of plant samples analyzed by Matos et al. [23] was smaller than that used in earlier studies [20, 21, 22], and did not include two important VNTRs: 002 and 077, which are designated as “cold Motif C and D” by Matos et al. [23]. In order to supplement the results of Matos et al. [23], we further analyzed changes in VNTRs through insect transmission and showed that some changes occurred even in the supposedly “cold Motifs”.

## Materials and Methods

### Psyllid and plant preparation

Wataru Ashihara, who worked in National Institute of Fruit Tree Science, received permission from the owner of the orchard to collect sample as well as the previous study [24]. A *Diaphorina citri* population originating from the Amami-ôshima Island of Kagoshima Prefecture, Japan was maintained on potted trees of orange jessamine, *Murraya paniculata* (L.) Jack (Rutaceae). To detect psyllid infection with ‘*Ca*. L. asiaticus’, PCR was conducted with OI1/OI2c primer [25, 26] and DNA extracted from feeding plant during periodic intervals. Rough lemon seedlings (*Citrus jambhiri* Lush, approximately 40 cm in height), grafted with ‘*Ca*. L. asiaticus’-infected scions originating from the Ishigaki Island of Okinawa Prefecture, Japan were used as a source for pathogen acquisition by psyllids. Plants were maintained in a temperature-controlled greenhouse at 30 and 25°C during the day and night, respectively. Healthy seedlings of yuzu (*Citrus junos* Tanaka), tankan mandarin (*Citrus tankan* Hayata), unzoki (*Citrus keraji* Tanaka), and orange jessamine (*M*. *maniculata*), approximately 10 cm in height, were prepared as transmission recipient plants. To detect infection with ‘*Ca*. L. asiaticus’, PCR was also conducted with OI1/OI2c primer [25, 26] and DNA extracted from their plant during periodic intervals.

### Psyllid transmission experiment

All experiments using live *D*. *citri* were performed in growth chambers at 25°C with a 16L:8D photoperiod at the Kuchinotsu Citrus Research Station, NIFTS (Otsu 954, Kuchinotsu, Minamishimabara, Nagasaki 859–2501, Japan). For adult acquisition–adult transmission experimental tests, 10-day-old post-emerging ‘*Ca*. L. asiaticus’- negative adults were placed on a leaf of an infected rough lemon tree within a plastic tube (9 cm in length and 3 cm in diameter) for an acquisition feeding. After an acquisition access period (AAP) of 55 days, each adult individual was transferred to a healthy yuzu seedling for an inoculation access period (IAP) of 20 days.

**Fig 1:**
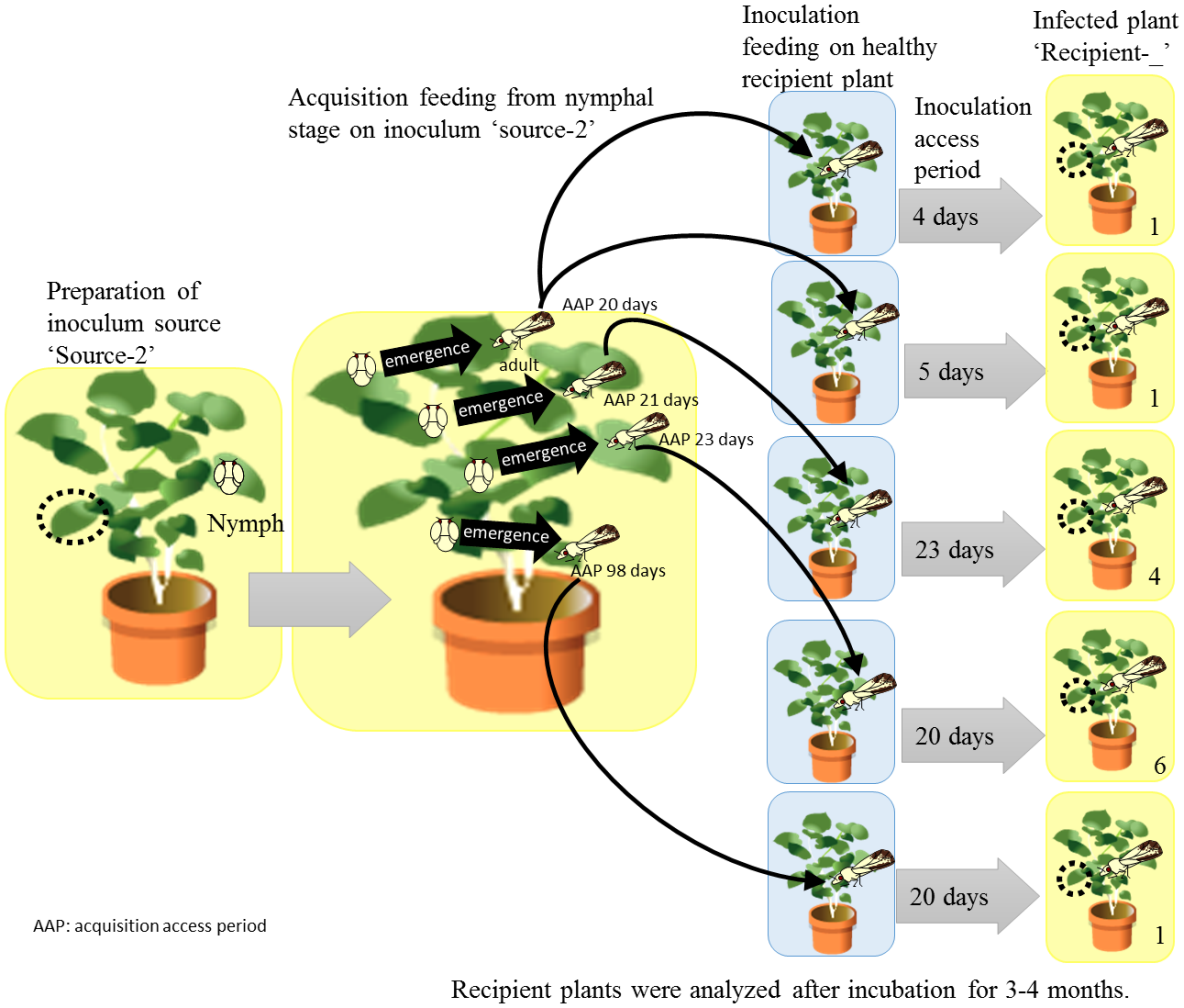
Schematic model of strategy of Table 2. The black circle indicates the area used for DNA extraction. Numbers at the lower right, which are x n, indicate the number of samples in Table 2.

As for nymphal acquisition–adult transmission tests, healthy fifth instars maintained on ‘*Ca*. L. asiaticus’-negative *M*. *paniculata* were transferred to an infected rough lemon tree with a fine brush. These nymphs became adults during the acquisition feeding. After an AAP of 20–98 days, each adult individual was transferred to a healthy yuzu, tankan mandarin, unzoki, or orange jessamine seedling for an IAP of 4–23 days. The number of insect used for each IAP is dictate in Fig 1. Recipient plants were maintained for 3–4 months in a temperature-controlled greenhouse until DNA exrtraction. After inoculation feeding, all psyllids were collected and preserved at –50°C until DNA extraction. For all psyllid transmission experiment, single psyllid was used in IAP per plant. Twenty-seven of 144 inoculative psyllids died during IAPs (mortality rate = 18.8%) and the pathogen was successfully transmitted to 42 of 144 recipient plants by psyllids (transmission rate = 29.2%). Inoculative psyllid DNA samples from which sufficient copies of ‘*Ca*. L. asiaticus’ genome were detected by quantitative real-time PCR assays [24] were used for the subsequent analysis of VNTRs of the “*Ca*, L, asiaticus” genome in psyllids.

### DNA extraction

Leaves were collected from the leaf midrib tissue of each leaf collected in citrus trees infected with ‘*Ca*. L. asiaticus’ was selected for DNA extraction. Total DNA was extracted with the DNeasy plant minikit (Qiagen, Valencia, CA) according to the manufacturer’s instructions.

In order to extract DNA from psyllids, the body was dissected into the anterior part (head and prothorax), which includes salivary glands, and the posterior part (mesothorax, metathorax, and abdomen) using a disposable razor. Total DNA was purified from each part of a single psyllid using the DNeasy Blood and Tissue Kit (Qiagen, Tokyo, Japan) and a plastic homogenizer pestle (As One, Tokyo, Japan) according to the manufacturer’s instructions, and eluted in 100 μL of AE buffer provided in the kit. PCR amplification was performed using each part of a single psyllid as template.

### Primers and PCR

Four loci within the ‘*Ca*. L. asiaticus’ genome containing TACAGAA, CAGT, AGACACA, and TTTG motifs [20, 21, 27] were examined in this study. These motifs had been previously designated as 001, 002, 005, and 077, respectively, [21]. The fragments containing these loci were amplified by PCR using the primer sets listed in Table 1, as previously reported [21]. Additionally, another forward primer was designed in order to be easy to count the motif of VNTR ‘001’ by using a program available on the Primer3 website (http://frodo.wi.mit.edu/primer3/) (Table 1).

**Table 1:** Primers for characterization of variable tandem repeats in four loci of the genome of '*Ca*. Liberibacter asiaticus' strains.

Motif	Sequence	Genome position	Primers' seqoence 5'-3'	Reference	Amplicon size(bp)
001	TACAGGA	255591–255646	(+)ggtgaattaggatggaaatgc	[21]	1159
			(-)tgaagtagctctgcaatatctga		
			(-)tgcctcttctagggcacaa	this paper	The primer was used for only sequence.
002	CAGT	537729–537760	(+)ttgataatatagaaagaggcgaagc	[21]	510
			(-)tccatacccaaaagaaaagca		
005	AGACACA	354493–354527	(+)attgaaggacgaaaccgatg	[21]	598
			(-)tcccaaggttttcaaattgc		
077	TTTG	655277–655332	(+)tgactgatggcaaaagatgg	[21]	528
			(-)agacacgccaaacaaggaat		

PCR was performed using a GeneAmp PCR System 9700 (Applied Biosystems, Foster City, CA, USA) in a 20-μL reaction mixture containing 1 μL of DNA template, 0.1 μM of each primer, 200 μM dNTP mixture, 1× PCR buffer, and 2.5 units of Ex Taq DNA polymerase Hot Start Version (TaKaRa, Shiga, Japan). The thermal cycling conditions were as follows: initial denaturation at 92°C for 2 min; 35 cycles of denaturing at 92°C for 30 s, annealing at 54°C for 30 s, and extension at 72°C for 1 min. Amplified PCR products were separated by electrophoresis in a 1.5% (wt/vol) agarose gel in Tris-boric acid EDTA buffer. Before direct sequencing, the PCR products were extracted from the gel slices using a QIAquick Gel Extraction Kit (Qiagen) according to the manufacturer’s instructions.

### Direct sequencing and data analysis

The nucleotide sequences of the DNA fragments were obtained by directly sequencing both strands of the purified PCR products using the dideoxynucleotide triphosphate (ddNTP) termination method [28] with ABI 3130 sequencer (Applied Biosystems, Foster City, CA, USA). The number of repetitions for each VNTR was manually counted from the sequence data.

## Results

### Change in VNTRs through psyllid transmission

Some changes were confirmed in the profiles of the tandem repeats with psyllid transmission (Table 2, Fig 1). Changes in VNTR ‘001’ occurred with psyllid transmission in two different citrus hosts (yuzu and unzoki). In addition, changes in VNTR ‘077’ were observed in psyllid transmission to orange jessamine (*Murraya paniculata*), a citrus relative (Table 2).

**Table 2:** Analysis of the stability of tandem repeats through nymphal acquisition–adult transmission experiments.

		VNTR					
Sample ID	Organism^d^	001	002	005	077	Acquisition access period (days)	Inoculation access period (days)
Source-2^a^	Rough lemon	14	8	8	9		
Recipient-4^b^	Yuzu	14	8	8	9	20	5
Recipient-5	Yuzu	14	8	8	9	20	4
Recipient-6	Unzoki	13 ^c^	8	8	9	23	20
Recipient-8	Unzoki	14	8	8	9	23	20
Recipient-9	Unzoki	14	8	8	9	23	20
Recipient-12	Unzoki	14	8	8	9	23	20
Recipient-13	Unzoki	15	8	8	9	23	20
Recipient-17	Unzoki	14	8	8	9	23	20
Recipient-18	Tankan mandarin	14	8	8	9	98	20
Recipient-19	Yuzu	14	8	8	9	21	23
Recipient-20	Yuzu	15	8	8	9	21	23
Recipient-21	Yuzu	14	8	8	9	21	23
Recipient-22	Orange jessamine	14	8	8	8	21	23

^a^‘*Ca*. L. asiaticus’-infected greenhouse grown rough lemon tree used as inoculum source for nymphal acquisition–adult transmission of the pathogen.

^b^Recepor plants that became infected upon psyllid transmission of the bacterium from 'Source-2' plant.

^c^Underline shows varied number of VNTR compared with that from 'Source-2' plant.

^d^Rough lemon, *Citrus jambhiri*; yuzu, *Citrus junos*; unzoki, *Citrus keraji*; tankan mandarin, *Citrus tankan*; orange jessamine, *Murraya paniculata*.

**Fig 2:**
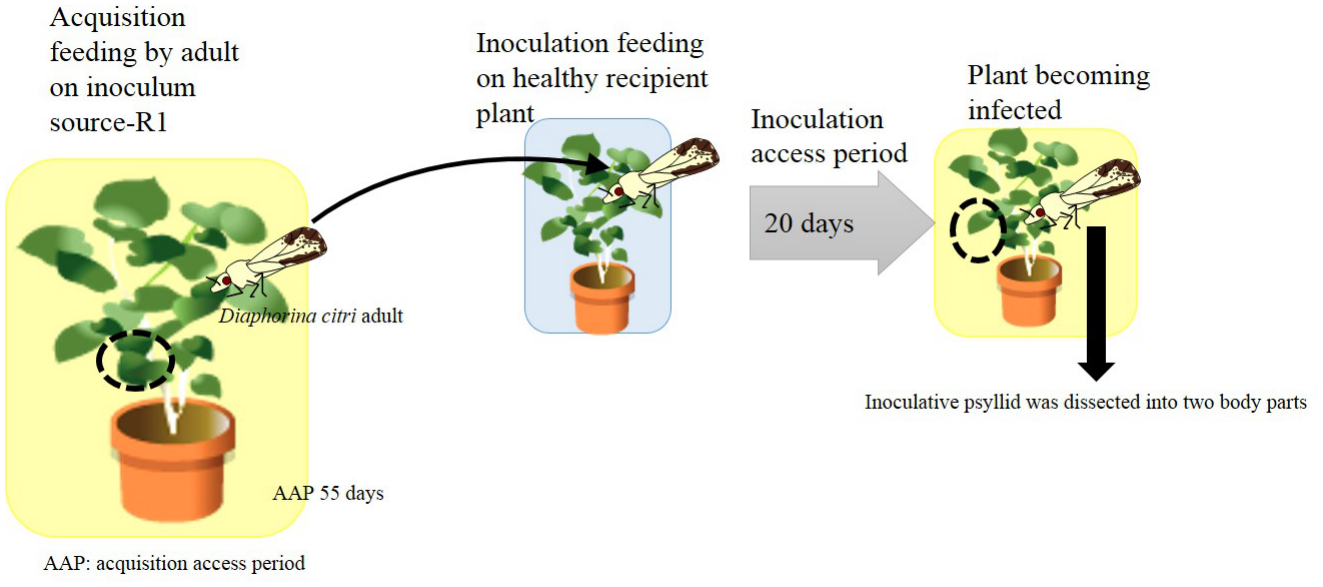
Schematic model of strategy of Table 3. The black circle indicates the area used for DNA extraction. Numbers at the lower right, which are x n, indicate the number of samples in Table 3.

Changes in VNTRs in the ‘*Ca*. L. asiaticus’ genome occurred while the bacterium was in the psyllid body (Table 3, Fig 2). These VNTR changes in the psyllid body might have contributed to the changes of VNTRs in the ‘*Ca*. L. asiaticus’ genome in the recipient plant in some cases. For example, the profile of VNTRs of ‘*Ca*. L. asiaticus’ in one yuzu recipient plant (sample ID: Recipient-Y17) perfectly matches the VNTRs of ‘*Ca*. L. asiaticus’ in the psyllid body parts (Table 3).

**Table 3:** Analysis of the stability of tandem repeats through adult acquisition–adult transmission experiments.

		VNTR					
Sample ID	Organism^g^	001	002	005	077	Acquisition access period (days)	Inoculation access period (days)
Source-R1^a^	Rough lemon	14	8	8	9		
Vector-head^b^-1^e^	Psyllid	14	8	8	9	55	
Vecor-abdomen^c^-1	Psyllid	14	8	8	9	55	
Recipient^d^-Y1	Yuzu	14	8	8	9		20
Vector-head-4	Psyllid	14	8	8	9	55	
Vecor-abdomen-4	Psyllid	14	8	8	9	55	
Recipient-Y4	Yuzu	14	9 ^f^	9	9		20
Vector-head-6	Psyllid	14	8	8	9	55	
Vecor-abdomen-6	Psyllid	14	9	8	9	55	
Recipient-Y6	Yuzu	14	8	8	9		20
Vector-head-8	Psyllid	14	8	8	9	55	
Vecor-abdomen-8	Psyllid	14	8	8	9	55	
Recipient-Y8	Yuzu	14	8	8	9		20
Vector-head-12	Psyllid	14	8	8	9	55	
Vecor-abdomen-12	Psyllid	14	8	8	9	55	
Recipient-Y12	Yuzu	14	8	8	9		20
Vector-head-13	Psyllid	14	8	8	9	55	
Vecor-abdomen-13	Psllid	15	8	8	9	55	
Recipient-Y13	Yuzu	15	8	8	9		20
Vector-head-15	Psyllid	14	8	8	9	55	
Vecor-abdomen-15	Psyllid	14	8	8	9	55	
Recipient-Y15	Yuzu	14	8	8	9		20
Vector-head-16	Psyllid	14	8	8	9	55	
Vecor-abdomen-16	Psyllid	14	8	8	9	55	
Recipient-Y16	Yuzu	14	8	8	9		20
Vector-head-17	Psyllid	15	8	8	9	55	
Vecor-abdomen-17	Psyllid	15	8	8	9	55	
Recipient-Y17	Yuzu	15	8	8	9		20
Vector-head-20	Psyllid	14	8	8	9	55	
Vecor-abdomen-20	Psyllid	14	8	8	9	55	
Recipient-Y20	Yuzu	14	8	8	9		20
Vector-head-21	Psyllid	14	8	8	9	55	
Vecor-abdomen-21	Psyllid	14	8	8	9	55	
Recipient-Y21	Yuzu	14	8	8	9		20
Vector-head-24	Psyllid	14	8	8	9	55	
Vecor-abdomen-24	Psyllid	14	8	8	9	55	
Recipient-Y24	Yuzu	14	8	8	9		20
Vector-head-27	Psyllid	14	8	8	9	55	
Vecor-abdomen-27	Psyllid	14	8	8	9	55	
Recipient-Y27	Yuzu	14	8	8	9		20
Vector-head-30	Psyllid	14	8	8	9	55	
Vecor-abdomen-30	Psyllid	14	8	8	9	55	
Recipient-Y30	Yuzu	14	8	9	9		20
Vector-head-11	Psyllid	14	8	8	9	55	
Vecor-abdomen-11	Psyllid	14	8	8	9	55	
Recipient-Y11	Yuzu	ND	ND	ND	ND		20
Vector-head-29	Psyllid	ND	ND	ND	ND	55	
Vecor-abdomen-29	Psyllid	ND	ND	ND	ND	55	
Recipient-Y29	Yuzu	16	8	8	9		20

^a^‘*Ca*. L. asiaticus’-infected greenhouse grown rough lemon (Source-R1) used as an inoculum source for psyllid transmission of the pathogen.

^b^Anterior part (including head and prothorax) of each psyllid individual after acquisition feeding on source-R1 plant and inoculation feeding on recipient yuzu seedling.

^c^Posterior part (including mesothorax, metathorax, and abdomen) of each psyllid individual after acquisition feeding on source-R1 plant and inoculation feeding on recipient yuzu seedling.

^d^Recipient plants that became infected upon psyllid transmission of the bacterium from the source plant 'Source-R1'.

^e^Last number between the psyllid as vector and the recipient plant was one-to-one correspondence, respectively.

^f^Underline shows varied number of VNTR compared with that from source plant ‘Source-R1’.

^g^Psyllid, *Diaphorina citri*; yuzu, *Citrus junos*.

However, VNTRs of ‘*Ca*. L. asiaticus’ in the body parts of psyllids were not always related to those of ‘*Ca*. L. asiaticus’ in the recipient plants. For example, three VNTRs of ‘*Ca*. L. asiaticus’ in one a yuzu recipient plant (sample ID: Recipient-Y4) were different from those of the source plant, whereas all four VNTRs of ‘*Ca*. L. asiaticus’ in the body parts of the vector were unchanged. Such discrepancy was also observed in the transmission test using sample ID Vector-abdomen-6 (psyllid abdomen) and sample ID Recipient-Y6 (recipient), as well as in the test using sample ID Vector-head-13 (psyllid head) and sample ID Recipient-Y13 (recipient).

**Fig 3:**
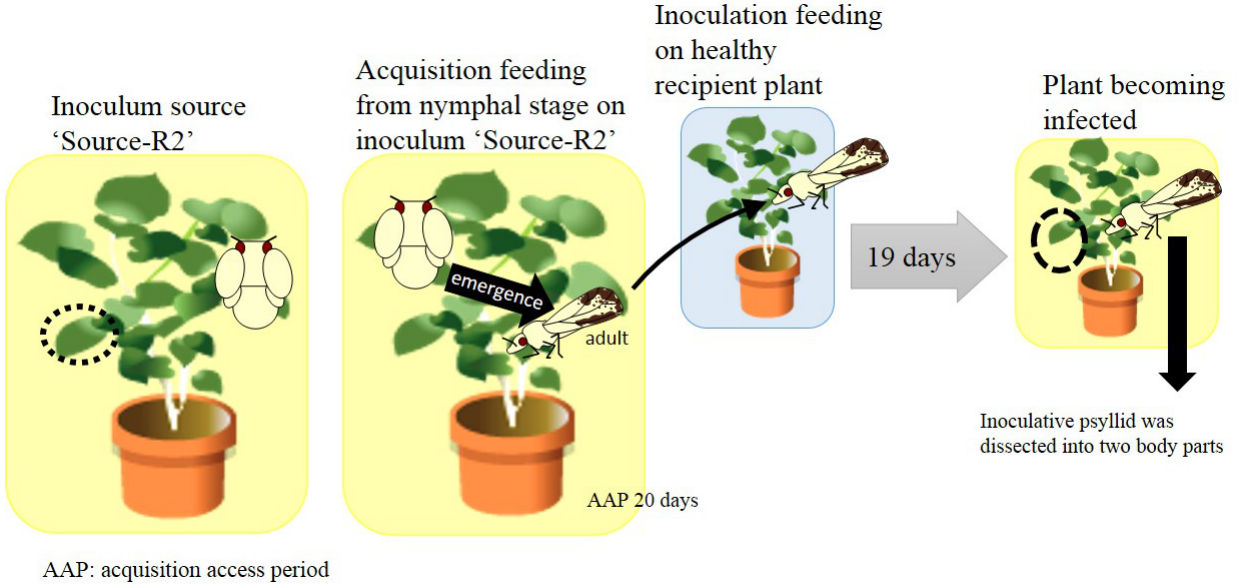
Schematic model of strategy of Table 4. The black circle indicates the area used for DNA extraction. Numbers at the lower right, which are x n, indicate the number of samples in Table 4.

In Table 3, we show that VNTRs change after psyllid transmission with AAP and IAP of 55 and 20 days, respectively. Subsequently, we attempted psyllid transmission with a shorter AAP and IAP, and found that the four VNTRs did not change with shorter periods: AAP and IAP of 20 and 19 days, respectively (Table 4, Fig 3).

**Table 4:** Analysis of the stability of tandem repeats through nymphal acquisition–adult transmission of the pathogen from yuzu infected with ‘*Ca*. L. asiaticus’ for a short period.

		VNTR					
Sample ID	Organism	001	002	005	077	Acquisition access period (days)	Inoculation access period (days)
Sorce-R2^a^	Yuzu	14	8	8	9	20	19
Vector-head^b^-10^e^	Citrus psyllid	14	8	8	9	20	
Vecor-abdomen^c^-10	Citrus psyllid	14	8	8	9	20	
Recipient^d^-Y10	Yuzu	14	8	8	9		19
Vector-head-12	Citrus psyllid	14	8	8	9	20	
Vecor-abdomen-12	Citrus psyllid	14	8	8	9	20	
Recipient-Y12	Yuzu	14	8	8	9		19
Vector-head-13	Citrus psyllid	14	8	8	9	20	
Vecor-abdomen-13	Citrus psyllid	14	8	8	9	20	
Recipient-Y13	Yuzu	14	8	8	9		19
Vector-head-14	Citrus psyllid	14	8	8	9	20	
Vecor-abdomen-14	Citrus psyllid	14	8	8	9	20	
Recipient-Y14	Yuzu	14	8	8	9		19
Vector-head-15	Citrus psyllid	14	8	8	9	20	
Vecor-abdomen-15	Citrus psyllid	14	8	8	9	20	
Recipient-Y15	Yuzu	14	8	8	9		19
Vector-head-31	Citrus psyllid	14	8	8	9	20	
Vecor-abdomen-31	Citrus psyllid	14	8	8	9	20	
Recipient-Y31	Yuzu	14	8	8	9		19
Vector-head-45	Citrus psyllid	14	8	8	9	20	
Vecor-abdomen-45	Citrus psyllid	14	8	8	9	20	
Recipient-Y45	Yuzu	14	8	8	9		19
Vector-head-48	Citrus psyllid	14	8	8	9	20	
Vecor-abdomen-48	Citrus psyllid	14	8	8	9	20	
Recipient-Y48	Yuzu	14	8	8	9		19

^a^‘*Ca*. L. asiaticus’-infected greenhouse grown yuzu (Sorce-R2) used as an inoculum source for psyllid transmission of the pathogen.

^b^Same number between the psyllid as vector and the recipient plant was one-to-one correspondence, respectively.

^c^Anterior part (including head and prothorax) of each psyllid individual after acquisition feeding on source-R2 plant and inoculation feeding on recipient yuzu seedling.

^d^Posterior part (including mesothorax, metathorax, and abdomen) of each psyllid individual after acquisition feeding on source-R2 plant and inoculation feeding on recipient yuzu seedling.

^e^Recipient plants that became infected upon psyllid transmission of the bacterium from the source Sorce-R2 plant.

## Discussion

This study suggested that psyllid transmission contributes to changes in the four VNTRs of ‘*Ca*. L. asiaticus’, and that both AAPs and IAPs by psyllids are involved. It was also suggested that longer acquisition/inoculation access would result in more changes in the four VNTRs, as significant differences in the frequency of alteration of tandem repeats among the four VNTRs were observed using direct sequencing. In this study, the most frequently varied tandem number among the four VNTRs was ‘001’, which changed in six samples in this study, and the VNTR ‘005’ locus was the second most frequently changed VNTR. In contrast, changes in VNTRs ‘002’ and ‘077’ were rarely seen in this study. These differences agree with Nei's measure of genetic diversity for each VNTR that was calculated among Japanese ‘*Ca*. L. asiaticus’ strains [21]. However, variability analysis of VNTRs with graft transmission is still insufficient because of the lack of specimens.

Matos et al. [23] indicated that the numbers of tandem repeats in the four loci tested displayed highly distinguishable “signature profiles” for the two Florida-type ‘*Ca*. L. asiaticus’ haplotype groups. However, the strain used in this study did not look like either of those haplotypes. The whole genome sequence of the strain used in this study also contained unique features [29]. The two haplotypes enumerated by Matos et al. [23] might show different trends when Japanese ‘*Ca*. L. asiaticus’ isolates are used for genetic analysis.

To our knowledge, this is the first report of a change in the tandem repeat VNTR ‘077’ after psyllid transmission. The results of this study suggested that the four VNTRs often change after psyllid transmission.

Folimonova et al. [30] indicated that real-time quantitative PCR assays of nonsymptomatic tissue located next to asymptomatic area for different hosts showed that this tissue sometimes did and sometimes did not contain detectable amounts of the bacterium [30]. Inoue et al. [24] suggested that longer AAPs result in higher percentages of ‘*Ca*. L. asiaticus’-positive psyllids, and that adult psyllids, after acquisition feeding in the nymph stage, enhanced proliferation and efficient transmission of the bacterium [24]. Growth efficiency might also affect stability of the four VNTRs in the bacterium.

The results of this study contribute to the understanding of relationships among different ‘*Ca*. L. asiaticus’ strains that are isolated from different fields and orchards, and might be used to examine effective transmission methods (by vegetative propagation or by psyllid transmission).
